# Misconceptions and Integration

**Published:** 2015-10

**Authors:** SARA MORTAZ HEJRI, AZIM MIRZAZADEH, MOHAMMAD JALILI

**Affiliations:** 1 Department of Medical Education, Tehran University of Medical Sciences, Tehran, Iran;; 2 Department of Internal Medicine, Department of Medical Education, Tehran University of Medical Sciences, Tehran, Iran;; 3 Department of Emergency Medicine, Department of Medical Education, Tehran University of Medical Sciences, Tehran, Iran

**Keywords:** Medical Education, Curriculum, Reform, Integration

## Abstract

**Introduction:**

Pervasive beliefs regarding curricular reform and integration have flourished among medical students, faculty members and medical school administrators. These concepts have extensively impacted the reform process, sometimes by resisting the reforms and sometimes by diverting the curriculum from its planned objectives. In the current paper, we have tried to address the challenges of integration in MD program by looking at the existing literature and the experience of the international universities.

**Methods:**

We collected the questions frequently asked during the curricular reform process. We, then, evaluated them, and selected 5 main ideas. In order to find their answers, we searched the literature using these keywords: integration, reform, and undergraduate medical curriculum.

**Results:**

The findings are discussed in five sections: 1) Reform is not equivalent to integration, 2) Integration can be implemented in both high school and graduate programs, 3) Organ-system based integration is not the only method available for integration, 4) Integration of two phases (basic sciences and physiopathology) can be considered but it is not mandatory, 5) Integration does not fade basic sciences in favor of clinical courses.

**Conclusion:**

It seems that medical education literature and prior experience of the leading universities do not support most of the usual concepts about integration. Therefore, it is important to consider informed decision making based on best evidence rather than personal opinions during the curricular reform process.

## Introduction

In recent years, in tandem with several curricular reform initiatives in medical universities, pervasive beliefs have flourished among medical students, faculty members and school administrators. These deeply-rooted and widely-distributed ideas have extensively impacted the reform process, sometimes by causing resistance against change and sometimes by diverting it from its planned objectives. However, in many cases the existing literature or the experience of other universities around the world does not support these ideas.

Because a significant portion of these beliefs deal with “integration”, in the current paper we have tried to address the challenges of integration in the MD program by looking at the existing literature and the experience of the international universities, while the basic concepts of integration such as its definitions, types and procedure have not been discussed here and readers are referred to the relevant literature (1-5).

The authors have been involved in the process of curricular reform of MD program and, hence, have participated in various sessions and group discussions. Several questions and challenges have been frequently raised in these sessions. These points have been collected and 5 main concepts that could have a considerable effect on the integration and reform process were selected as follows:

1. The main focus of curricular reform is integration of courses.
2. Integration is only suitable for Graduate Entry programs.
3. The only available method for integrating courses is organizing them into organ-system based blocks.
4. Combination of two pre-clinical phases (basic sciences and pathophysiology) should be considered for integration.
5. The integration results in fading away of basic sciences in favor of clinical sciences.

To explore the aforementioned arguments, manual searching, electronic searching of online databases such as PubMed, Elsevier, EBSCO and Google Scholar, and searching the websites of different universities were done. The keywords included integration, reform, and undergraduate medical curriculum. The findings were classified into the following parts corresponding to the five above-mentioned points.

1. **Reform is** **not equivalent to integration. **
It is noteworthy that sometimes when one is speaking of reform, it is considered synonymous with integration; this is true to the extent that in some universities the only change actually being made is integration and most of the “reformed” programs in the country are commonly referred to as “integrated curricula” among faculty members and students. This is even the case in some of the published articles. Even the concept of integration has been trivialized to horizontal integration of courses in the basic science phase (histology, embryology, physiology, etc.)(6) or pathophysiology phase (pathology, pharmacology, internal medicine and pathophysiology) (7, 8) and little attention has been paid to the vertical integration.
However, contrary to the common belief, integration is only one facet of curricular reform that targets the curriculum structure. There are, yet, many other challenging issues which should be considered and although, given the amount of available resources, it is not always likely to conduct a fundamental renewal, having a comprehensive image of all aspects of the curriculum including teaching methods and assessment techniques can be helpful. Before starting the new program in Tehran University of Medical Sciences (TUMS), the traditional curriculum was evaluated by four large independent studies. In the final comprehensive evaluation report, a list of strengths and weaknesses of the traditional program was presented (9). This report highlighted the importance of dealing with other aspects of reform which was translated into the TUMS reform vision statement (10).
2. **Integration can be implemented in both high school entry and graduate entry programs. **
  One of the common arguments is that integration is only applicable in the graduate entry programs, in which students have already learned the majority of the basic sciences topics and are ready to understand the integrated courses. Therefore, since the majority of medical students in Iran are admitted directly from high school, it is not appropriate to integrate the curriculum. Pertaining to this viewpoint, three issues must be considered:
  - The concept of integration is associated with how different topics are connected to each other. It considers the organization and layout of the courses rather than their content. In other words, integration does not mean eliminating the educational content. What often gives the impression that the integration reduces the delivered content to students is that a material repeatedly presented in various courses within a traditional program can be aggregated after the integration through coordination among teachers of different disciplines.
  - In many universities with graduate entry programs, the previous field of study is not substantially related to medical courses. Even graduates of disciplines such as psychology, management, linguistics, economics, and, music are admitted while their courses are naturally not dominated by the basic medical sciences and so only a minimum of training in courses such as chemistry, physics and biology is required (11).
  - The integrated curriculum can also be seen in programs in which students from high school are admitted. In countries such as Australia and United Kingdom, where common model of student admission from high school has been followed for several years, there are integrated forms of medical curricula (12).
3. **Organ-system** **based integration is not the only method available for integration. **
  To operationalize the concept of integration, common themes that relate different content areas should be found. Three approaches are briefly discussed below:
  - **Organ-system based integration**: The first and the most common pattern of integration is the use of body systems (such as cardiovascular system) as the common theme for organizing the curriculum content (2). All elements of the system, previously taught separately in different disciplines such as anatomy, embryology, physiology, histology, pathology, pharmacology and pathophysiology, are collated and put together into one single block (3).
  - **Life-cycle based integration**: In this approach, topics related to the structure and function of the human body and concepts related to the physical and mental growth are presented according to their chronology in modules of pregnancy, embryonic stages, infancy, childhood, adolescence, adulthood and, old age. Examples include Plymouth University in England and University of New South Wales in Australia (13, 14).
  - **Key-concepts or health-problems based integration**: This approach, also called multi-system based integration (2), is especially adapted in problem-based learning curricula, like McMaster University (15). Medical curriculum of Liverpool University with 58 modules such as hypertension, infection, anesthesia, and fracture (16), and also medical curriculum of the University of California San Francisco with modules such as cancer and metabolism in addition to modules that are intended for organs and life cycle are representatives of this category (17). In Iran, Shahroud University of Medical Sciences has similar modules, namely, neoplasia and the host defense in addition to the organ-system blocks (18).
4. **Integration** **of two pre-clinical phases (basic sciences and pathophysiology) is possible, but not mandatory. **
  It should be mentioned that the basic sciences (two and a half years) and pathophysiology phases (one year) of the national medical curriculum in Iran, together, are almost equivalent to the pre-clinical phase (two years) of other universities, usually delivered in one of the two following types:
In some curricula, the first year of the pre-clinical phase is devoted mainly to the normal structure and function of the human body (basic sciences such as anatomy, histology, physiology, and embryology) and the second year focuses mainly on the abnormal structure and function (pathophysiology, pathology, and microbiology). When the phase is offered as integrated, this general principle is preserved. In other words, normal and abnormal disciplines associated with different systems are offered in the integrated blocks of the first year and the second year, respectively. Examples include medical programs of the University of California, Los Angeles and Yale University (19-21). In this case, every organ-system is offered twice during the pre-clinical phase (22) (Table 1).
  **Table 1:** The schematic design of pre-clinical phase in an integrated curriculum in which the basic sciences (normal structure and function) and the pathophysiology (abnormal structure and function) are separated

	The first semester				The second semester			
**The first year**	Cardiovascular 1	Respiratory 1	Gastrointestinal 1	Endocrine 1	Urinary 1	Reproductive 1	Neural 1	Musculoskeletal 1
	Anatomy	Anatomy	Anatomy	Anatomy	Anatomy	Anatomy	Anatomy	Anatomy
	Physiology	Physiology	Physiology	Physiology	Physiology	Physiology	Physiology	Physiology
	Histology	Histology	Histology	Histology	Histology	Histology	Histology	Histology
	Embryology	Embryology	Embryology	Embryology	Embryology	Embryology	Embryology	Embryology
**The second year**	Cardiovascular 2	Respiratory 2	Gastrointestinal 2	Endocrine 2	Urinary 2	Reproductive 2	Neural 2	Musculoskeletal 2
	Pathophysiology	Pathophysiology	Pathophysiology	Pathophysiology	Pathophysiology	Pathophysiology	Pathophysiology	Pathophysiology
	Pathology	Pathology	Pathology	Pathology	Pathology	Pathology	Pathology	Pathology
	Immunology	Immunology	Immunology	Immunology	Immunology	Immunology	Immunology	Immunology
	Pharmacology	Pharmacology	Pharmacology	Pharmacology	Pharmacology	Pharmacology	Pharmacology	Pharmacology
In other curricula, as implemented in the University of Manchester and the University of California, San Francisco, all materials related to a system, whether normal or abnormal, are integrated in one integrated block (17, 23, 24). In this case, every system is presented only once during the pre-clinical phase (Table 2).
  **Table 2:** The schematic design of pre-clinical phase in an integrated curriculum in which the basic sciences (normal structure and function) and the pathophysiology (abnormal structure and function) are combined.

	The first semester		The second semester	
**The first year**	Cardiovascular	Respiratory	Gastrointestinal	Endocrine
	Anatomy	Anatomy	Anatomy	Anatomy
	Physiology	Physiology	Physiology	Physiology
	Histology	Histology	Histology	Histology
	Embryology	Embryology	Embryology	Embryology
	Pathophysiology	Pathophysiology	Pathophysiology	Pathophysiology
	Pathology	Pathology	Pathology	Pathology
	Immunology	Immunology	Immunology	Immunology
	Pharmacology	Pharmacology	Pharmacology	Pharmacology
**The second year**	Urinary	Reproductive	Neural	Musculoskeletal
	Anatomy	Anatomy	Anatomy	Anatomy
	Physiology	Physiology	Physiology	Physiology
	Histology	Histology	Histology	Histology
	Embryology	Embryology	Embryology	embryology
	Pathophysiology	Pathophysiology	Pathophysiology	Pathophysiology
	Pathology	Pathology	Pathology	Pathology
	Immunology	Immunology	Immunology	Immunology
	Pharmacology	Pharmacology	Pharmacology	Pharmacology
  In summary, two phases of basic sciences and pathophysiology do not necessarily need to be integrated. Especially for universities that have traditionally offered the two phases separately, it should be noted that this change alone is a major step in changing the layout and requires careful preparation.
5. **Integration does not fade** **basic sciences away in favor of clinical sciences. **
According to the results of a study in which the perception of students in the traditional and the new curricula about the importance of basic sciences were evaluated, the students from the new curriculum were more satisfied with how they had been taught basic sciences (25). However, one of the concerning issues following the integration for faculty members of basic science departments is fading of basic sciences courses. This arises from two reasons.
  - The first problem is the prevailing attitude of the administrative bodies that fuel this argument by delegating the major role of decision making on different aspects of integration to clinicians.
  - The second problem stems from the tendency of basic scientists to give long lectures dealing with great details, which in most cases are not useful in medical profession (26). The presumption that if all the content is not offered in the class, students will not be able to learn it, can be seen in both traditional and integrated curriculum and can lead students to memorize basic sciences and do not try to develop their understanding of the fundamentals of medicine.
However, basic sciences is what differentiates medical doctors from other healthcare providers and is very helpful in the patient management, particularly the "complex" cases (27). Medical students in the integrated curriculum can learn the fundamental concepts of basic sciences, and in association with clinical issues, set the appropriate ground for enhancing their clinical decision making and reasoning skills. In order to emphasize the role of basic sciences in the integrated curriculum, some suggestions are offered here.
  - When designing the new integrated curriculum, it seems more logical to accept the central role of basic sciences faculty members and take advantage of advisory comments of clinical faculty members.
  - Faculty members of basic sciences should believe that it is not possible to transfer their whole knowledge to students, and this is basically not required either and could eventually lead to an opposite effect. It would be beneficial to use the opportunities offered in the integrated curriculum and encourage students to generate hypotheses and apply scientific methods in order to develop lifelong learning skills (27).
  - Some universities have offered basic sciences courses in the clinical phase. These include presenting “back to basic sciences" at Mayo Medical School (27, 28), holding PBL sessions at Australian National University (29) and using electronic modules at Leiden University Medical Center, the Netherlands (30).

## Conclusions

As medical education curricula undergo reform in the country, some beliefs have been formed among administration staff, faculty members and students on how the reform and integration are being designed and implemented. Although the origin of some of these ideas is unknown, the reform process throughout the country has been affected by them.

In this paper, we collected a variety of frequently asked questions regarding integration of the MD curriculum, and after selecting five main concepts, we searched through literature, evidence and experiences of other universities in order to find the right answers. It seems that medical education literature and prior experience of the leading universities do not support most of the common beliefs about integration. The findings stress the importance of informed decision making based on best evidence rather than personal opinions during the curricular reform process. 

## References

[1] Harden RM (2000). The integration ladder: a tool for curriculum planning and Evaluation. Med Educ.

[2] Bandaranayake RC (2011). The integrated medical curriculum.

[3] Dent JA, Harden RM (2013). A Practical Guide for Medical Teachers.

[4] Ashoorion V, Sharif M (2011). Trend of Recent Changes in Medical Education Curriculum in the World: The Location of Iranian Medical Education Curriculum. Iranian Journal of Medical Education.

[5] Yamani N, Shater Jalali M (2012). Curriculum Integration, with Emphasis on Integration in Medical Education. Iranian Journal of Medical Education.

[6] Sum S, Alinegad S, Rastgar Z, Tashakkori F, Khani A, Pourghasem M (2012). Basic Science Lecturer’s Perspectives on Integration in Babol University of Medical Sciences. Iranian Journal of Medical Education.

[7] Jalilian N, Jalilian N, Rezaei M, Deh Haghi AA (2011). Evaluating the satisfaction of Kermanshah University of Medical Sciences students of Physiopathology course integration. Journal of Horizons of Medical Education Development.

[8] Karimian Z, Amini M, Sagheb MM (2011). Evaluation of horizontal integration pathophysiology of medical school program from students’ viewpoint. Journal of Horizons of Medical Education Development.

[9] Comprehensive Evaluation of TUMS MD program [Internet].

[10] TUMS MD Program Vision Statement [Internet].

[11] MD Program Application Process FAQs [Internet].

[12] Elliott MK (1999). Are we going in the right direction? A survey of the undergraduate medical education in Canada, Australia and United Kingdom from a general practice perspective. Med Teach.

[13] MD program of Plymouth University [Internet].

[14] McNeil HP, Hughes CS, Toohey SM, Dowton SB (2006). An innovative outcomes-based medical education program built on adult learning principles. Med Teach.

[15] MD program [Internet].

[16] Jones GL, Walley T, Bligh J (1997). Integrating clinical pharmacology in a new problem based medical undergraduate curriculum. Br J Clin Pharmacol.

[17] Muller JH, Jain S, Loeser H, Irby DM (2008). Lessons learned about integrating a medical school curriculum: perceptions of students, faculty and curriculum leaders. Med Educ.

[18] MD program [Internet].

[19] Wilkes MS, Usatine R, Slavin S, Hoffman JR (1998). Doctoring: University of California, Los Angeles. Acad Med.

[20] MD program David Geffen School of Medicine [Internet].

[21] MD program [Internet].

[22] Schmidt H (1998). Integrating the teaching of basic sciences, clinical sciences, and bio-psycho-social issues. Acad Med.

[23] O'Neill PA (1998). Problem-based learning alongside clinical experience: Reform of the Manchester. Education for Health.

[24] Mennin SP, Kalishman S (1998). Student assessment. Acad Med.

[25] Custers EJ, Ten Cate OH (2007). Medical clerks' attitudes towards the basic sciences: a longitudinal and a cross-sectional comparison between students in a conventional and an innovative curriculum. Med Teach.

[26] Cooke M, Irby DM, Sullivan W, Ludmerer KM (2006). American medical education 100 years after the Flexner report. N Engl J Med.

[27] Grande JP (2009). Training of physicians for the twenty-first century: Role of the basic sciences. Med Teach.

[28] Gregory JK, Lachman N, Camp CL, Chen LP, Pawlina W (2009). Restructuring a basic science course for core competencies: An example from anatomy teaching. Med Teach.

[29] Gatenby PA, Martin R (2009). Development of basic medical sciences in a new medical school with an integrated curriculum: The ANU experience. Med Teach.

[30] Dubois EA, Franson KL (2009). Key steps for integrating a basic science throughout a medical school curriculum using an e-learning approach. Med Teach.

